# Neuroprotective effects of human umbilical cord-derived mesenchymal stem cells on periventricular leukomalacia-like brain injury in neonatal rats

**DOI:** 10.1186/s41232-016-0032-3

**Published:** 2017-01-16

**Authors:** Chikako Morioka, Motohiro Komaki, Atsuko Taki, Izumi Honda, Naoki Yokoyama, Kengo Iwasaki, Sachiko Iseki, Tomohiro Morio, Ikuo Morita

**Affiliations:** 1 Department of Pediatrics and Developmental Biology, Graduate School of Medical and Dental Sciences, Tokyo Medical and Dental University, 1-5-45 Yushima, Bunkyo-Ku, Tokyo, 113-8510 Japan; 2 Department of Nanomedicine (DNP), Graduate School of Medical and Dental Science, Tokyo Medical and Dental University, 1-5-45 Yushima, Bunkyo-Ku, Tokyo, 113-8510 Japan; 3 Department of Cellular Physiological Chemistry, Graduate School of Medical and Dental Science, Tokyo Medical and Dental University, 1-5-45 Yushima, Bunkyo-Ku, Tokyo, 113-8510 Japan; 4 Department of Molecular Craniofacial Embryology, Graduate School of Medical and Dental Science, Tokyo Medical and Dental University, 1-5-45 Yushima, Bunkyo-Ku, Tokyo, 113-8510 Japan; 5 Life Science Laboratory, Research and Development Center, Dai Nippon Printing Co., Ltd., 1-1-1 kaga-cho, Shinjuku-ku, Tokyo, 162-8001 Japan

**Keywords:** Mesenchymal stem cells, Regeneration, Inflammation, Neonatal brain injury

## Abstract

**Background:**

Periventricular leukomalacia (PVL) is a type of multifactorial brain injury that causes cerebral palsy in premature infants. To date, effective therapies for PVL have not been available. In this study, we examined whether mesenchymal stem cells (MSCs) possess neuroprotective property in a lipopolysaccharide (LPS)-induced neonatal rat PVL-like brain injury.

**Methods:**

Human umbilical cord-derived MSCs (UCMSCs) were used in this study. Four-day-old rats were intraperitoneally injected with LPS (15 mg/kg) to cause the PVL-like brain injury and were treated immediately after the LPS-injection with UCMSCs, conditioned medium prepared from MSCs (UCMSC-CM) or interferon-gamma (IFN-γ)-pretreated MSC (IFN-γ-UCMSC-CM). To assess systemic reaction to LPS-infusion, IFN-γ in sera was measured by ELISA. The brain injury was evaluated by immunostaining of myelin basic protein (MBP) and caspase-3. RT-PCR was used to quantitate pro-inflammatory cytokine levels in the brain injury, and the expression of tumor necrosis factor-stimulated gene-6 (TSG-6) or indoleamine 2,3-dioxygenase (IDO) to evaluate anti-inflammatory or immunomodulatory molecules in UCMSCs, respectively. A cytokine and growth factor array was employed to investigate the cytokine secretion profiles of UCMSCs.

**Results:**

Elevated serum IFN-γ was observed in LPS-infused rats. The expression of IL-6, tumor necrosis factor-alpha (TNF-α), IL-1ß, and monocyte chemoattractant protein-1 (MCP-1) were increased in the brain by LPS-infusion in comparison to saline-infused control. LPS-infusion increased caspase-3-positive cells and decreased MBP-positive area in neonatal rat brains. A cytokine and growth factor array demonstrated that UCMSCs secreted various cytokines and growth factors. UCMSCs significantly suppressed IL-1ß expression in the brains and reversed LPS-caused decrease in MBP-positive area. UCMSC-CM did not reverse MBP-positive area in the injured brain, while IFN-γ-UCMSC-CM significantly increased MBP-positive area compared to control (no treatment). IFN-γ-pretreatment increased TSG-6 and IDO expression in UCMSCs.

**Conclusion:**

We demonstrated that bolus intraperitoneal infusion of LPS caused PVL-like brain injury in neonatal rats and UCMSCs infusion ameliorated dysmyelination in LPS-induced neonatal rat brain injury. Conditioned medium prepared from IFN-γ-pretreated UCMSCs significantly reversed the brain damage in comparison with UCMSC-CM, suggesting that the preconditioning of UCMSCs would improve their neuroprotective effects. The mechanisms underline the therapeutic effects of MSCs on PVL need continued investigation to develop a more effective treatment.

## Background

Periventricular leukomalacia (PVL) is a serious neonatal complication, in which periventricular white matter brain is injured in a fetus or neonate, which leads to cerebral palsy. Therefore, PVL exerts a critical influence over the prognosis of a premature infant. PVL has a multifactorial etiology that includes intrauterine infection/inflammation, neural cell and vascular prematurity, ischemia/reperfusion, and microglial activation. It causes dysmyelination due to disturbances in oligodendrocyte lineage development [1]. Despite the high prevalence of the disease, no effective therapies are available.

Mesenchymal stem cells (MSCs) are somatic tissue-derived cells with self-renewability and the potential to differentiate into various cell linages. Recently, therapeutic effects of MSCs for neurological diseases, such as hypoxic-ischemic encephalopathy, cerebral infarction, Alzheimer’s disease, and Parkinson’s disease, have been reported [2–6]. Several groups, including ours, have shown that MSCs differentiate into neurofilament (NFM)-positive neuron-like cells, glial fibrillary acidic protein (GFAP)-positive astrocyte-like cells, and 2′,3′-cyclic-nucleotide 3′-phosphodiesterase (CNPase)-positive oligodendrocyte-like cells [7–11]. MSCs are also known to secrete growth factors, cytokines, and chemokines for a wide range of activities, such as vascular endothelial growth factor receptor (VEGF) for anti-apoptosis; VEGF, and fibroblast growth factors (FGF-2) for angiogenesis; human tumor necrosis factor-stimulated gene-6 (TSG-6) and prostaglandin E2 (PGE-2) for anti-inflammation; indoleamine 2,3-dioxygenase (IDO),transforming growth factor (TGF-β), inducible nitric oxide synthase (iNOS) for immunomodulation; TGF-β, hepatocyte growth factor (HGF) anti-scarring; stromal cell-derived factor-1 (SDF-1) for chemoattraction, homing to injured tissues, and support of growth and differentiation of stem and progenitor cells in lesions [12–14]. IDO plays a critical role in immunomodulation by MSCs in its stimulation of regulatory T cells [15]. The expression of IDO in MSCs is upregulated under hypoxic conditions or with IFN-γ pretreatment [16, 17]. TSG-6 is one of the genes activated in response to inflammatory stimuli, such as TNF-α, IL-1, LPS, and TGF-β. Therapeutic activities of MSCs are known to depend in part on TSG-6 in animal disease models, such as those of cerebral ischemia, autoimmune encephalomyelitis, diabetes, and peritoneal adhesions [18].

In addition, Chang CP et al. found that MSCs changed their properties with culture conditions or in response to microenvironments such as inflammation and hypoxia [19]. Similar observation was also found that the effects of MSCs were reinforced by crosstalk with inflammatory stimulants and other affected cells [20].

In this study, we developed a neonatal rat with PVL-like brain injury by intraperitoneal administration of lipopolysaccharide (LPS) and examined the therapeutic effect of UCMSCs. Myelin basic protein (MBP) is a major structural component of myelin and is exclusively expressed in myelinating oligodendrocytes in brain. We found that LPS-infusion led to increase in the number of caspase-3-positive cells and reduction in MBP-positive area and that UCMSCs ameliorated LPS-induced reduction of MBP. In contrast, UCMSC-CM did not reverse MBP-positive area in the injured brain. We also found that conditioned medium prepared from IFN-γ-pretreated UCMSC (IFN-γ-UCMSC-CM) ameliorated reduction of MBP and that various cytokines, including TSG-6 or IDO were increased in interferon-gamma (IFN-γ)-simulated UCMSCs.

## Methods

### Animal

Postnatal day 4 (P4) Sprague-Dawley rats weighting 9–10 g were provided by Sankyo Labo Service Corporation, Inc. (Tokyo, Japan). All experimental procedures were performed in accordance with guidelines of the Institutional Animal Care and Use Committee, and were approved by the Animal Research Committee, Graduate School of Medical and Dental Science, Tokyo Medical and Dental University (0140112C). Animals were maintained in a temperature-controlled animal care facility under a 12-h light/dark cycle with a constant supply of food and water.

### Cell culture

Human umbilical cords-derived MSCs (UCMSCs) were obtained from Promo Cell GmbH (Heidelberg, Germany) or The Institute of Medical Science, The University of Tokyo. Two different sources of UCMSCs were examined their phenotypes by FACS analyses and by the differentiation assay. We confirmed that both cells exhibited MSC phenotype based on their cell surface marker expression and their differentiation capability into osteoblasts, chondroblasts, and adipocytes. All experimental procedures were approved by the Institutional Ethics Committee, Graduate School of Medical and Dental Science, Tokyo Medical and Dental University. The MSCs used in all experiments were from passage 4–7. The cells were cultured in a humidified incubator at 37 °C with 5% CO_2_ in α-minimum essential medium (α-MEM) (Life Technologies Japan, Tokyo, Japan) supplemented with 10% heat-inactivated fetal bovine serum, 1% antibiotic-antimycotic solution (Life Technologies Japan), and 1% L-glutamine (Life Technologies Japan). The medium was changed every 3 days.

### Preparation of the rat PVL model

To generate an inflammation-related PVL-like brain injury in rats, pups were injected with LPS (*Escherichia coli*, 055:B5, Sigma-Aldrich, St. Louis, MO, USA) intraperitoneally on day 4 at a dose of 15 mg/kg in 50 μl saline using a 32-gauge needle. The control pups received equal amounts of saline intraperitoneally. The dose schedule was based on a preliminary study. After this procedure, pups were housed with dams for 48 h or 8 d, as required for further experiments. Systemic reaction to the LPS-injection was monitored by IFN-γ in sera. The brain injury was assessed by proinflammatory chtokine level and MBP area.

### Preparation and treatment of MSC and MSC-conditioned medium (MSC-CM)

The UCMSCs were cultured in α-MEM supplemented with 10% fetal bovine serum to 70% confluency, when the medium was replaced with serum-free low-glucose DMEM (Life Technologies Japan). The UCMSCs were cultured in serum-free medium for 48 h to collect conditioned medium. Then, the conditioned medium was concentrated 20-fold using an Amicon Ultra with a 10-kDa cut-off (Millipore, Billerica, MA) and stored at 4 °C for the following experiment.

We injected saline or LPS into rats on postnatal day 4. In the LPS group, animals were randomly assigned to one of the two groups to receive intraperitoneal injections of UCMSCs, UCMSC-CM, or serum-free DMEM as a control medium, immediately after LPS-administration. Each pup was administered 1 × 10^6^ cells in 100 μl of serum-free DMEM for the UCMSC treatment or 100 μl of UCMSC-CM on 4 consecutive days. The concentrated conditioned medium was prepared with the secretions from an approximately equivalent number of UCMSCs (1 × 10^6^ MSCs). For the pretreatment of UCMSCs by IFN-γ, UCMSCs were treated with recombinant human IFN-γ (10 ng/ml, R&D Systems, Minneapolis, MN, USA). For the RT-PCR analysis, total RNA was extracted from UCMSCs 24 h after IFN-γ stimulation. The conditioned medium was collected and concentrated for treatment of LPS induced brain injury at 48 h after IFN-γ stimulation.

### Tissue preparation

Animals were deeply anesthetized by pentobarbital sodium and transcardially perfused with 0.1 M phosphate-buffered saline (PBS, pH 7.4), followed by 4% paraformaldehyde. Brains were removed from coronal sections at approximately the bregma on P12 and postfixed for 1 day in the same fixative, followed by 7-day incubation in 70% ethanol at 4 °C, and then dehydrated by graded alcohol concentration and embedded in paraffin. Coronal sections were cut at 6-μm thickness using a bright rotary microtome (Leica RM 2235 Wetzlar, Germany), and four sequential sections were collected.

### Immunohistochemical and histological analyses

To evaluate white matter injury, we investigated by immunohistochemistry the expression of MBP, which is a mature oligodendrocyte marker. For the immunohistochemistry, paraffin-embedded sections were deparaffinized in xylol, rehydrated with decreasing alcohol concentrations, and heat-unmasked for 15 min in 10-mM citrate buffer (pH 6.0). To inhibit endogenous peroxidase activity, sections were treated with 0.3% hydrogen peroxide and then blocked for 30 min with PBS containing 2% normal horse serum. Sections were incubated with primary antibodies, mouse monoclonal MBP (1:500; SMI-94R, Covance, Princeton, NJ), and caspase-3 antibody (1:1000; Cell Signaling Technology Japan), overnight at 4 °C in a wet chamber. After extensive washes in PBS, sections were further incubated with biotinylated secondary antibody for 30 min at room temperature, followed by application of peroxidase-coupled avidin-biotin complex (ABC Kit, Vector Laboratories, Burlingame, CA). The immunoreaction products were visualized with 3, 3-diaminobenzidine (DAB, Sigma-Aldrich), then counterstained with methyl green. All images were blinded and obtained on a light microscope (CTR 5000B, Leica, Wetzlar, Germany). We measured the positive area by ImageJ software (version 1.27z, National Institute of Health, Bethesda, MD, USA).

### RNA extraction and real-time RT-PCR analysis

In order to investigate the mechanisms underlying UCMSC or UCMSC-CM effects, we examined the inflammatory cytokine and chemokine expression levels in P6 rat brains using real-time RT-PCR. For total RNA isolation, the periventricular white matter tissue was removed from gross coronal sections from approximately −1 to +1 mm bregma. The tissue was immediately submerged in RNAlater solution (Invitrogen, Carlsbad, CA, USA) and stored at 4 °C until use. Then, the tissue was homogenized by Polytron (Central Scientific Commerce, Tokyo, Japan) with Isogen (Nippon Gene Co. Tokyo, Japan).

To investigate the secretion profiles of UCMSCs influenced by IFN-γ, we checked the expression of TSG-6 or IDO in MSCs with and without stimulation by IFN-γ. Total RNA from cell monolayers was extracted using an RNeasy Mini Kit (Qiagen, Venlo, Netherlands) after incubation for 24 h in the presence of IFN-γ.

About 1 μg of total RNA per sample was used to synthesize double-stranded cDNA by reverse transcription (First Strand cDNA Synthesis Kit for RT-PCR, AMV, Roche Diagnostics, Basel, Switzerland).

Real-time amplification was performed with SYBER GREEN I Master (Roche Diagnostics) and analyzed on a Light Cycler System (Light Cycler　480 II, Roche Diagnostics).

The assay was performed in three technical replicates for each biological sample.

Results of the targeted mRNA were normalized against the internal control, GAPDH.

### Cytokine array

An analysis of cytokines in conditioned media was performed using the Proteome Profiler Human Cytokine Array Panel A (R&D Systems) according to the manufacturer’s instructions. A growth factor analyses was performed using the Ray Bio C-Series Human Growth Factor Antibody Array C1 (Ray Biotech, Norcross, GA, USA). Colorimetric analysis using a luminescent image analyzer (Image Quant LAS 4000 mini) was used to quantify the intensity of each membrane dot, which allowed for the assessment of growth factor content in the UCMSC-CM. The conditioned media was used after 20-fold concentration.

### ELISA

Rat serum IFN-γ (Rat IFN-γ ELISA kit; R&D Systems) was detected with a commercially available ELISA kit following the procedures described by the manufacturer.

### Statistical analysis

All statistical analyses were performed using JMP software (SAS Institute Inc., NC, USA). Data are presented as the mean ± SEM. Multiple group comparisons were performed by Dunnett’s test. A two-tailed paired *t* test was used for pairwise comparisons. Statistical significance was defined as *p* < 0.05.

## Results

### LPS-induced rat PVL-like brain injury model

We examined whether intraperitoneal administration of LPS caused white matter degradation in neonatal rats. According to our preliminary experiments (data not shown), the optimal dose (15 mg/kg) of LPS was used to cause white matter degradation with less than 80% mortality in four-day-old rats in this study.

**Fig. 1:**
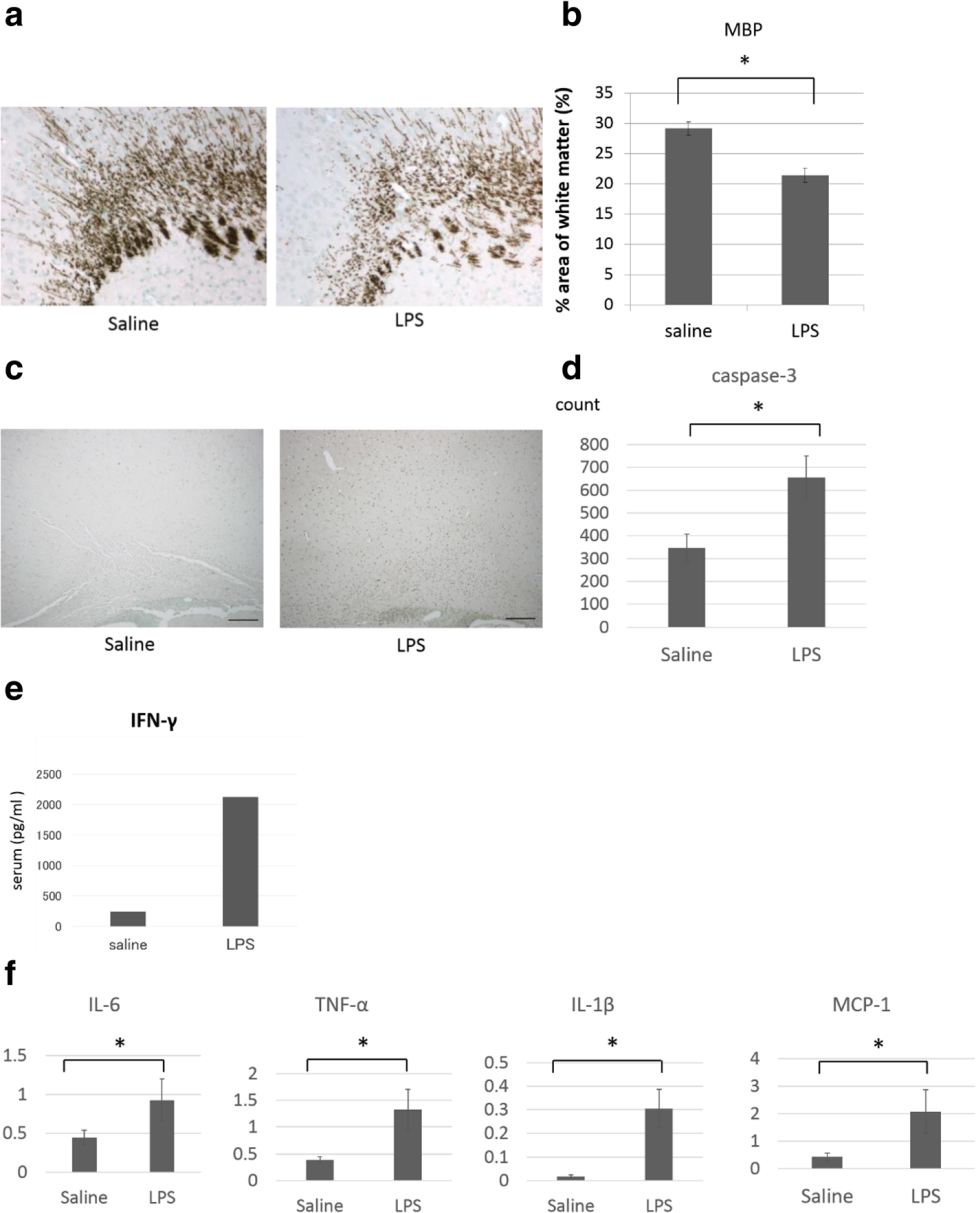
LPS-induced white matter brain injury. **a** The area of white matter was evaluated by MBP staining on postnatal day 12. Representative images from each group. From the left, the saline group and LPS group are shown. *Scale bar*: 100 μm. **b** Quantitative analysis of the MBP-positive areas in each group. Four sections were evaluated per pup. *N* = 21 and 18 in each group, values indicate means ± S.E. *, *p* < 0.05; assessed by *t* test. **c** The areas of white matter was evaluated by caspase-3 staining on postnatal day 12. Representative images of each group. From the left, the saline group and LPS group are shown. *Scale bar*: 100 μm. **d** Quantitative analysis of the caspase-3-positive area in each group. Four sections were evaluated per pup. *N* = 8 per group, values indicate means ± S.E. *; *p* < 0.05; assessed by *t* test. **e** IFN-γ levels in sera were assayed by ELISA at 6 h after LPS injection and compared with levels in the saline group. **f** The mRNA levels of pro-inflammatory cytokines and chemokines in brain tissues at 48 h after LPS treatment were determined by quantitative real-time reverse-transcription polymerase chain reaction (qRT-PCR). Relative mRNA expression levels of IL-6, TNF-α, IL-1β, and MCP-1 compared with levels in the saline group. *N* = 5, values indicate means ± S.E. *, *p* < 0.05; assessed by *t* test

In brain sections, immunostaining of MBP in brain sections showed coarse-staining and a reduction in the positive-staining area (Fig. 1a, b), and caspase-3-positive cells were increased in the LPS group, (Fig. 1c, d), indicating that the administration of LPS successfully caused white matter brain injury in neonatal rats. In order to examine the systemic reaction of the rats to LPS-infusion, we also measured the serum level of IFN-γ by ELISA and found that IFN-γ was significantly increased in the LPS group (Fig. 1e). In the brain, the expression of IL-6, tumor necrosis factor-alpha (TNF-α), IL-1ß, and monocyte chemoattractant protein-1 (MCP-1) were increased by LPS-infusion in comparison to saline-infused control (Fig. 1f).

### The effect of UCMSCs on LPS-induced brain injury

**Fig. 2:**
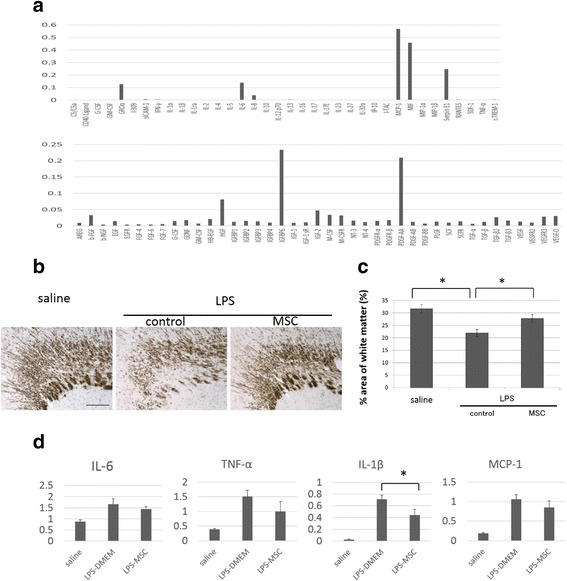
The effect of UCMSCs on LPS-induced brain injury. **a** Graphs showed the relative intensity ratios of growth factors found in the conditioned media to that of the provided positive control. **b** The area of white matter was evaluated by MBP staining after MSC treatment on postnatal day 12. Representative images from each group. From the left, the saline group, LPS-control group and LPS + MSC group are shown. *Scale bar*: 100 μ. **c** Quantitative analysis of the MBP-positive areas in each group. Four sections were evaluated per pup. *N* = 8 in each group, values indicate means ± S.E. *, *p* < 0.05; assessed by *t* test. **d** The mRNA levels of pro-inflammatory cytokines and chemokines in brain tissues at 48 h after MSC treatment were determined by qRT-PCR. Relative mRNA expression levels of IL-6, TNF-α, IL-1β, and MCP-1 compared with levels in the saline group. *N* = 5, values indicate means ± S.E. *, *p* < 0.05; assessed by *t* test

First, we confirmed that UCMSCs were positive for CD44, CD73, CD90, and CD105, and negative for CD11b, CD31, CD34, CD45, and HLA-DR by flow cytometric analyses. Differentiation of UCMSCs into osteoblasts, adipocytes, or chondrocytes in the induction medium was also confirmed (data not shown). Next, we examined a cytokine and growth factor array and confirmed that UCMSCs secreted various cytokines and growth factors such as MCP-1, MIF, IGFBP-6, and PDGF (Fig. 2a).

In order to investigate whether UCMSCs affected LPS-induced white matter brain injury in neonatal rats, UCMSCs were administrated intraperitoneally immediately after the LPS injection. We found that the administration of UCMSCs significantly reversed the MBP-positive area on P12 rats in the LPS-MSC group in comparison with that in the LPS control group, implying that UCMSCs-infusion significantly improved myelination (Fig. 2b, c). To investigate the effect of UCMSCs on inflammatory cytokine expression in the lesion, the mRNA expression of inflammatory cytokines in rat brains were analyzed by real-time RT-PCR 48 h after UCMSC-infusion. The administration of UCMSCs tended to decrease the proinflammatory cytokine levels. Among them, the effect of UCMSCs on IL-1β suppression was statistically significant (Fig. 2d).

### The effect of UCMSC-CM on LPS-induced brain injury

**Fig. 3:**
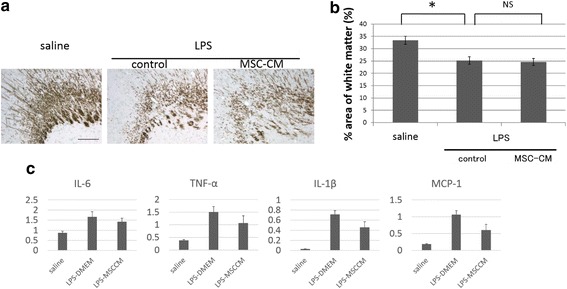
The effect of UCMSC-CM on LPS-induced brain injury. **a** The area of white matter was evaluated by MBP staining after MSC treatment on postnatal day 12. Representative images from each group. From the left, the saline group, LPS-control group and LPS + MSC group are shown. *Scale bar*: 100 μm. **b** Quantitative analysis of the MBP-positive areas in each group. Four sections were evaluated per pup. *N* = 8 in each group, values indicate means ± S.E. *, *p* < 0.05; assessed by *t* test. **c** The mRNA levels of pro-inflammatory cytokines and chemokines in brain tissues at 48 h after MSC-CM treatment were determined by qRT-PCR. Relative mRNA expression levels of IL-6, TNF-α, IL-1β, and MCP-1 compared with levels in the saline group. *N* = 5 , values indicate means ± S.E. *, *p* < 0.05; assessed by *t* test

We then examined whether UCMSC-CM could also suppressed LPS-induced white matter brain injury. UCMSC-CM was administrated four consecutive days after the LPS-injection. Although a trend towards suppressed expression in the inflammatory cytokines levels were observed in the brain (Fig. 3c), UCMSC-CM did not reverse the MBP-positive area on P12 (Fig. 3a, b).

### The effect of preconditioning of UCMSCs on the LPS-induced white matter brain injury

**Fig. 4:**
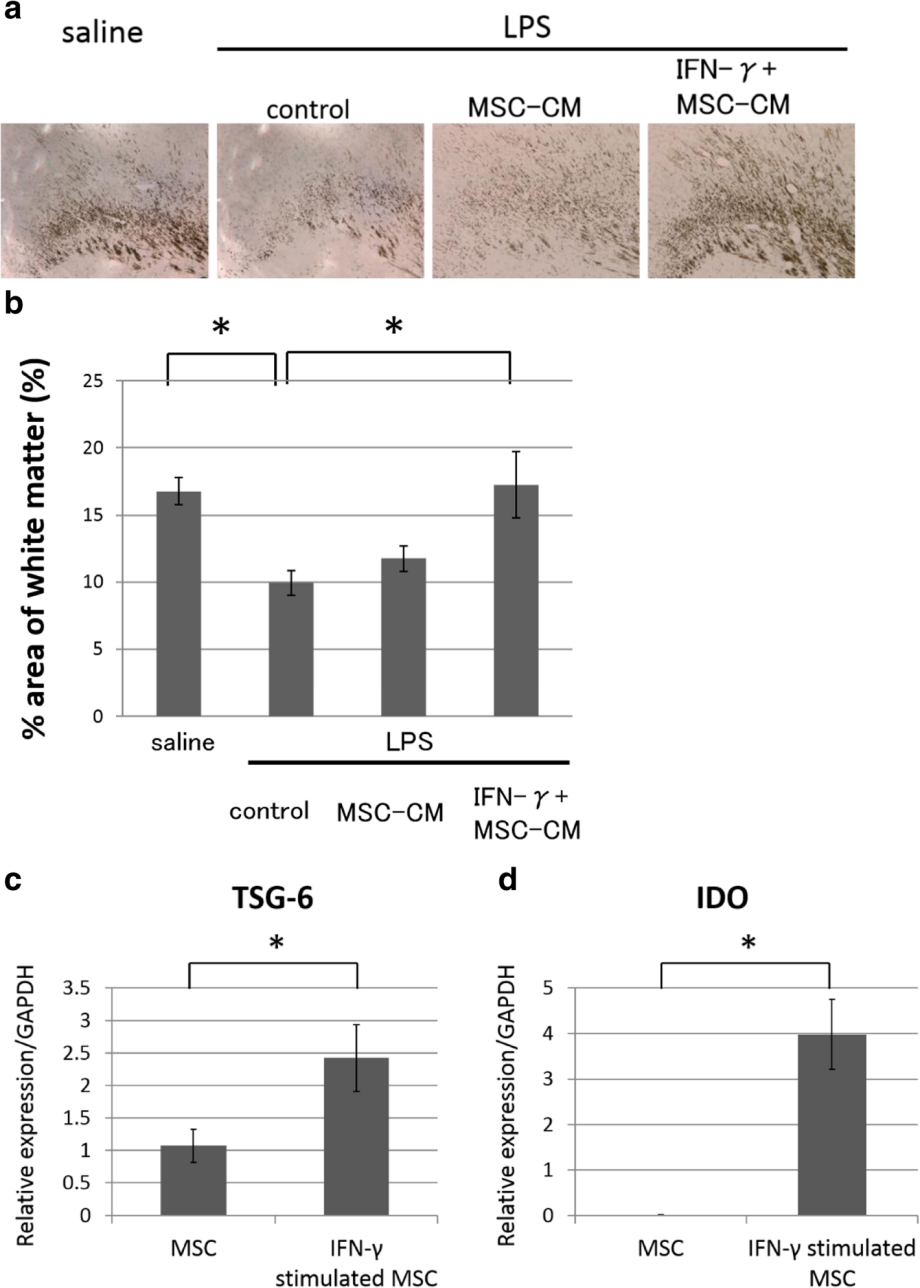
The effect of preconditioning of UCMSCs on the LPS-induced white matter brain injury. **a** The areas of white matter was evaluated by MBP staining on postnatal day 12. Representative images of each group. From the left, the saline group, LPS+ control group, LPS + MSC-CM group, and LPS + IFN-γ-MSC-CM group are shown. *Scale bar*: 100 μm. **b** Quantitative analysis of the MBP-positive area in each group. Four sections were evaluated per pup. *N* = 7 or 8 per group, values indicate means ± S.E. *; *p* < 0.05; assessed by Dunnett’s test. **c** Expression levels of TSG-6 and IDO in MSC stimulated with IFN-γ using quantitative RT-PCR. *, *p* < 0.05; assessed by *t* test

We next examined discrepancy in the neuroprotective effect between UCMSCs and UCMSC-CM in this model. Previously, it has been reported that MSCs exposed to inflammatory stimuli exhibited anti-inflammatory effects [21]. Folkerth et al. have reported that macrophage-produced IFN-γ plays a role in PVL [22]. We found that the serum level of IFN-γ was significantly increased in the LPS group (Fig. 1e). We examined whether IFN-γ-preconditioning of UCMSCs could improve the effect of UCMSC-CM in LPS-induced dysmyelination. IFN-γ-UCMSC-CM was administrated four consecutive days immediately after the LPS-injection. In contrast to UCMSC-CM, IFN-γ-UCMSC-CM effectively reversed the MBP area on P12 (Fig. 4a, b), suggesting that preconditioning of USMSCs with IFN-γ reinforced neuroprotecitive effects of UCMSCs. Our preliminary experiments showed that no big differences in cytokine secretion profile between UCMSC-CM and IFN-γ-UCMSC-CM. We then investigated whether the expression of TSG-6, one of the most important anti-inflammatory molecules in MSCs was influenced by IFN-γ-pretreatment. Real-time RT-PCR analysis showed that the expression of TSG-6 markedly increased when UCMSCs were pretreated by IFN-γ for 24 h (Fig. 4c). Moreover, IDO expression in UXMSCs was also increased by IFN-γ-pretreatment (Fig. 4c).

## Discussion

The purpose of this study was to examine whether UCMSCs have a therapeutic effect on an LPS-induced rat PVL model. We generated the rat PVL-like brain injury model by intraperitoneal injection of high dose-LPS on P4, when oligodendrocyte progenitor cells proliferate in the periventricular region of the brain. Our results showed a decrease in MBP and increase of caspase-3 positive cells in this region and upregulation of pro-inflammatory cytokines after LPS injection, suggesting the abrogation of myelination because of brain inflammation. Moreover, intraperitoneally administrated MSCs abated dysmyelination in this model and suppressed LPS-induced IL-1β levels in the brain tissues. Previous reports have shown that MSCs improved hypoxia/ischemia-induced brain injury [23, 24]. However, the mechanisms that underlie the therapeutic effects of MSCs remain elusive. Our results suggest that the therapeutic effects of MSCs can be attributed in part to a reduction in local inflammation.

Previous reports showed that small numbers of the delivered MSCs appeared to reach the inflamed or damaged tissues, suggesting that the therapeutic action of MSCs might be related to their trophic mechanisms rather than to their ability to reach damaged tissues [25, 26]. We then examined whether factors secreted from MSCs could also improve LPS-induced brain injury. Contrary to our expectations, our results showed that MSC-CM did not suppress the MBPs degradation significantly. Intraperitoneally injected MSCs were recently reported to form aggregates in the peritoneum, where they produced immunoregulatory or anti-inflammatory molecules [27, 28]. In a preliminary experiment, when MSCs were intraperitoneally administrated after LPS injection, we observed that the luciferase labeled MSCs remained in intraperitoneal region during 14 days without migration to the region of brain injury. Whereas, in this study, MSC-CM prepared from the same number of MSCs used for the cell administration study did not show therapeutic effects.

Previously, Duijvestein et al. reported that MSCs exposed to inflammatory stimuli such as IFN-γ exhibit anti-inflammatory effects [21]. We assumed that the difference between the effects of MSCs and MSC-CM on myelination was due to pre-exposure of the MSCs to IFN-γ. We demonstrated that IFN-γ-MSC-CM improved MBP levels compared to the control, suggesting that IFN-γ-pretreatment could augment the therapeutic effects of MSCs. Our preliminary experiments showed that IFN-γ levels in the conditioned medium with or without IFN-γ-pretreat were not detectable, suggesting that the augmented therapeutic effects of MSCs were not attributed to carryover effects of IFN-γ. Intriguingly, we found that the expression of the anti-inflammatory factor TSG-6 and IDO was increased by IFN-γ stimulation. These results suggest that MSCs can produce a variety of anti-inflammatory factors following IFN-γ stimulation.

Recent reports have shown that the paracrine potential of MSCs can be enhanced by various pretreatments. Chen et al*.* reported that bone marrow derived MSC-CM, which was preactivated under radiation-induced inflammatory conditions, showed dramatic improvements in damaged intestines and in survival of irradiated rats [29]. Moreover, preactivation of MSCs with TNF-α, IL-1β, and nitric oxide enhanced its paracrine effects on radiation-induced intestinal injury. Another report suggested that the immunosuppressive properties of MSCs were augmented by IFN-γ-pretreatment in vitro [30].

We demonstrated that MSCs improved LPS-induced brain injury and that IL-1β was significantly suppressed by MSC-treatment (Fig. 2d). However, it seems to be insufficient to explain the differences in neuroprotective effects between MSCs and MSC-CM since IL-1β tended to be suppressed after MSC-CM-treatment (Fig. 3c). It has been reported that therapeutic effects of MSCs were attributed to anti-inflammation, anti-apoptosis, anti-oxidization, angiogenesis, regulation of microglia activation, activation of oligodendrocyte differentiation. Further experiments are required to elucidate the underlying mechanism. Moreover, it is also interesting to employ behavior test to ask whether MSCs or IFN-γ-MSC-CM could improve cerebral palsy in our model.

## Conclusions

In conclusion, we firstly demonstrated that MSCs have therapeutic effects on LPS-induced PVL-like brain injury and these effects could be augmented under proper stimuli. The mechanisms underline the therapeutic effects of MSCs on PVL need continued investigation to develop more effective treatment.

## Abbreviations

CNPase: 2′,3′-cyclic-nucleotide 3′-phosphodiesterase
FGF-2: Fibroblast growth factors
GFAP: Glial fibrillary acidic protein
HGF: Hepatocyte growth factor
IDO: Indoleamine 2,3-dioxygenase
IDO: Indoleamine 2,3-dioxygenase
IFN-γ: Interferon-gamma
IFN-γ-UCMSC-CM: IFN-γ-pretreated MSC
iNOS: Inducible nitric oxide synthase
LPS: Lipopolysaccharide
MBP: Myelin basic protein
MCP-1: Monocyte chemoattractant protein-1
MSCs: Mesenchymal stem cells
NFM: Neurofilament
PBS: Phosphate-buffered saline
PGE-2: Prostaglandin E2
PVL: Periventricular leukomalacia
SDF-1: Stromal cell-derived factor-1
TGF-β: Transforming growth factor
TNF-α: Tumor necrosis factor-alpha
TSG-6: Tumor necrosis factor-stimulated gene-6
TSG-6: Tumor necrosis factor-stimulated gene-6
UCMSC-CM: Conditioned medium prepared from MSCs
UCMSCs: Umbilical cord-derived MSCs
VEGF: Vascular endothelial growth factor receptor
α-MEM: α-minimum essential medium

## References

[1] Volpe JJ, Kinney HC, Jensen FE, Rosenberg PA (2011). Reprint of “The developing oligodendrocyte: key cellular target in brain injury in the premature infant”. Int J Dev Neurosci.

[2] Chen A, Siow B, Blamire AM, Lako M, Clowry GJ (2010). Transplantation of magnetically labeled mesenchymal stem cells in a model of perinatal brain injury. Stem Cell Res.

[3] van Velthoven CT, Kavelaars A, Heijnen CJ (2012). Mesenchymal stem cells as a treatment for neonatal ischemic brain damage. Pediatr Res.

[4] Borlongan CV, Weiss MD (2011). Baby STEPS: a giant leap for cell therapy in neonatal brain injury. Pediatr Res.

[5] Titomanlio L, Kavelaars A, Dalous J, Mani S, El Ghouzzi V, Heijnen C, Baud O, Gressens P (2011). Stem cell therapy for neonatal brain injury: perspectives and challenges. Ann Neurol.

[6] van Velthoven CT, Sheldon RA, Kavelaars A, Derugin N, Vexler ZS, Willemen HL, Maas M, Heijnen CJ, Ferriero DM (2013). Mesenchymal stem cell transplantation attenuates brain injury after neonatal stroke. Stroke.

[7] Techawattanawisal W, Nakahama K, Komaki M, Abe M, Takagi Y, Morita I (2007). Isolation of multipotent stem cells from adult rat periodontal ligament by neurosphere-forming culture system. Biochem Biophys Res Commun.

[8] Borkowska P, Kowalska J, Fila-Danilow A, Bielecka AM, Paul-Samojedny M, Kowalczyk M, Kowalski J. Affect of antidepressants on the in vitro differentiation of rat bone marrow mesenchymal stem cells into neuronal cells. Eur J Pharm Sci. 2015;73. 10.1016/j.ejps.2015.03.016 25841360

[9] Li Z, Zhao W, Liu W, Zhou Y, Jia J, Yang L (2014). Transplantation of placenta-derived mesenchymal stem cell-induced neural stem cells to treat spinal cord injury. Neural Regen Res.

[10] Lo Furno D, Pellitteri R, Graziano AC, Giuffrida R, Vancheri C, Gili E, Cardile V (2013). Differentiation of human adipose stem cells into neural phenotype by neuroblastoma- or olfactory ensheathing cells-conditioned medium. J Cell Physiol.

[11] Bonnamain V, Thinard R, Sergent-Tanguy S, Huet P, Bienvenu G, Naveilhan P, Farges JC, Alliot-Licht B. Human dental pulp stem cells cultured in serum-free supplemented medium. Front Physiol. 2013;4. 10.3389/fphys.2013.00357 PMC3858652 24376422

[12] Bai L, Lennon DP, Caplan AI, DeChant A, Hecker J, Kranso J, Zaremba A, Miller RH (2012). Hepatocyte growth factor mediates mesenchymal stem cell-induced recovery in multiple sclerosis models. Nat Neurosci.

[13] Lee RH, Oh JY, Choi H, Bazhanov N (2011). Therapeutic factors secreted by mesenchymal stromal cells and tissue repair. J Cell Biochem.

[14] Roddy GW, Oh JY, Lee RH, Bartosh TJ, Ylostalo J, Coble K, Rosa RH, Prockop DJ (2011). Action at a distance: systemically administered adult stem/progenitor cells (MSCs) reduce inflammatory damage to the cornea without engraftment and primarily by secretion of TNF-alpha stimulated gene/protein 6. Stem Cells.

[15] Krampera M, Cosmi L, Angeli R, Pasini A, Liotta F, Andreini A, Santarlasci V, Mazzinghi B, Pizzolo G, Vinante F (2006). Role for interferon-gamma in the immunomodulatory activity of human bone marrow mesenchymal stem cells. Stem Cells.

[16] Croitoru-Lamoury J, Lamoury FM, Caristo M, Suzuki K, Walker D, Takikawa O, Taylor R, Brew BJ (2011). Interferon-gamma regulates the proliferation and differentiation of mesenchymal stem cells via activation of indoleamine 2,3 dioxygenase (IDO). PLoS One.

[17] Francois M, Romieu-Mourez R, Li M, Galipeau J (2012). Human MSC suppression correlates with cytokine induction of indoleamine 2,3-dioxygenase and bystander M2 macrophage differentiation. Mol Ther.

[18] Prockop DJ (2013). Concise review: two negative feedback loops place mesenchymal stem/stromal cells at the center of early regulators of inflammation. Stem Cells.

[19] Chang CP, Chio CC, Cheong CU, Chao CM, Cheng BC, Lin MT (2013). Hypoxic preconditioning enhances the therapeutic potential of the secretome from cultured human mesenchymal stem cells in experimental traumatic brain injury. Clin Sci (Lond).

[20] Gunn WG, Conley A, Deininger L, Olson SD, Prockop DJ, Gregory CA (2006). A crosstalk between myeloma cells and marrow stromal cells stimulates production of DKK1 and interleukin-6: a potential role in the development of lytic bone disease and tumor progression in multiple myeloma. Stem Cells.

[21] Duijvestein M, Wildenberg ME, Welling MM, Hennink S, Molendijk I, van Zuylen VL, Bosse T, Vos AC, de Jonge-Muller ES, Roelofs H (2011). Pretreatment with interferon-gamma enhances the therapeutic activity of mesenchymal stromal cells in animal models of colitis. Stem Cells.

[22] Folkerth RD, Keefe RJ, Haynes RL, Trachtenberg FL, Volpe JJ, Kinney HC (2004). Interferon-gamma expression in periventricular leukomalacia in the human brain. Brain Pathol (Zurich, Switzerland).

[23] Zhu LH, Bai X, Zhang N, Wang SY, Li W, Jiang L. Improvement of human umbilical cord mesenchymal stem cell transplantation on glial cell and behavioral function in a neonatal model of periventricular white matter damage. Brain Res. 2014;1563. 10.1016/j.brainres.2014.03.030 24680746

[24] Lee JA, Kim BI, Jo CH, Choi CW, Kim EK, Kim HS, Yoon KS, Choi JH (2010). Mesenchymal stem-cell transplantation for hypoxic-ischemic brain injury in neonatal rat model. Pediatr Res.

[25] Prockop DJ, Kota DJ, Bazhanov N, Reger RL (2010). Evolving paradigms for repair of tissues by adult stem/progenitor cells (MSCs). J Cell Mol Med.

[26] Ranganath SH, Levy O, Inamdar MS, Karp JM (2012). Harnessing the mesenchymal stem cell secretome for the treatment of cardiovascular disease. Cell Stem Cell.

[27] Bartosh TJ, Ylostalo JH, Bazhanov N, Kuhlman J, Prockop DJ (2013). Dynamic compaction of human mesenchymal stem/precursor cells into spheres self-activates caspase-dependent IL1 signaling to enhance secretion of modulators of inflammation and immunity (PGE2, TSG6, and STC1). Stem Cells.

[28] Sala E, Genua M, Petti L, Anselmo A, Arena V, Cibella J, Zanotti L, D’Alessio S, Scaldaferri F, Luca G (2015). Mesenchymal stem cells reduce colitis in mice via release of TSG6, independently of their localization to the intestine. Gastroenterology.

[29] Chen H, Min XH, Wang QY, Leung FW, Shi L, Zhou Y, Yu T, Wang CM, An G, Sha WH, et al. Pre-activation of mesenchymal stem cells with TNF-alpha, IL-1beta and nitric oxide enhances its paracrine effects on radiation-induced intestinal injury. Sci Rep. 2015;5. 10.1038/srep08718 PMC4346809 25732721

[30] Chinnadurai R, Copland IB, Patel SR, Galipeau J (2014). IDO-independent suppression of T cell effector function by IFN-gamma-licensed human mesenchymal stromal cells. J Immunol.

